# Retrospective analysis of a case series of patients with traumatic injuries to the craniocervical junction

**DOI:** 10.1590/S1679-45082016AO3396\

**Published:** 2016

**Authors:** Luiz Adriano Esteves, Andrei Fernandes Joaquim, Helder Tedeschi

**Affiliations:** 1 Universidade Estadual de Campinas, Campinas, SP, Brazil.; 2 Núcleo do Hospital de Força Aérea de São Paulo, São Paulo, SP, Brazil.

**Keywords:** Cervical vertebrae/injuries, Wounds and injuries/classification, Wounds and injuries/surgery, Wounds and injuries/therapy

## Abstract

**Objective:**

To evaluate the correlation between the treatment, the characteristics of the lesions and the clinical outcome of patients with traumatic injuries to the craniocervical junction.

**Methods:**

This was a retrospective study of patients treated conservatively or surgically between 2010 and 2013 with complete data sets.

**Results:**

We analyzed 37 patients, 73% were men with mean age of 41.7 years. Of these, 32% were submitted to initial surgical treatment and 68% received conservative treatment. Seven (29%) underwent surgery subsequently. In the surgical group, there were seven cases of odontoid type II fractures, two cases of fracture of posterior elements of the axis, one case of C1-C2 dislocation with associated fractured C2, one case of occipitocervical dislocation, and one case of combined C1 and C2 fractures, and facet dislocation. Only one patient had neurological *déficit* that improved after treatment. Two surgical complications were seen: a liquoric fistula and one surgical wound infection (reaproached). In the group treated conservatively, odontoid fractures (eight cases) and fractures of the posterior elements of C2 (five cases) were more frequent. In two cases, in addition to the injuries of the craniocervical junction, there were fractures in other segments of the spine. None of the patients who underwent conservative treatment presented neurological deterioration.

**Conclusion:**

Although injuries of craniocervical junction are relatively rare, they usually involve fractures of the odontoid and the posterior elements of the axis. Our results recommend early surgical treatment for type II odontoid fractures and ligament injuries, the conservative treatment for other injuries.

## RESUMO

**Objetivo:**

Avaliar a correlação entre o tratamento, as características das lesões e o resultado clínico em pacientes com lesões traumáticas na junção craniocervical.

**Métodos:**

Estudo retrospectivo de pacientes maiores de 18 anos tratados de forma conservadora ou cirúrgica, entre 2010 e 2013.

**Resultados:**

Foram analisados 37 pacientes, 73% eram do sexo masculino e a média de idade foi de 41,7 anos. Inicialmente 32% dos pacientes foram submetidos a tratamento cirúrgico, e 68% foram submetidos a tratamento conservador. Sete pacientes (29%) do grupo conservador foram submetidos posteriormente à cirurgia. No grupo cirúrgico, houve sete casos de fratura de odontóide tipo II, dois casos de fratura de elementos posteriores do áxis, um caso de luxação C1-C2, um caso de deslocamento occipito-cervical e um caso de fraturas de C1 e C2 e luxação facetária. Um paciente apresentava *déficit* neurológico, melhorando após o tratamento. Houve duas complicações pós-cirúrgicas, uma fístula liquórica e uma infecção de ferida operatória (reabordada). No grupo conservador, predominaram as fraturas do odontóide (oito) e dos elementos posteriores de C2 (cinco). Em dois casos, havia também fraturas em outros segmentos da coluna. Nenhum dos pacientes deste grupo apresentou deterioração neurológica.

**Conclusão:**

As lesões da junção craniocervical são raras, sendo mais frequentes as fraturas do odontóide e dos elementos posteriores do áxis. Nossos resultados recomendam o tratamento cirúrgico precoce para os pacientes com fraturas do odontóide tipo II e lesões ligamentares, e tratamento conservador para os demais pacientes.

## INTRODUCTION

Traumatic injuries of the craniocervical junction (CCJ) affect mostly young adults, and cause enormous physical, psychological and social consequences. While the frequency of spinal injuries is increasing due to the growing number of traffic accidents, the mortality has fallen mainly due to the improvement of the initial treatment.^(1-3)^

Traumatic injuries of the CCJ characteristically involve the skull base, the atlas and axis.^(3)^ They have a low prevalence compared with injuries of other spinal segments and they present unique characteristics, such as the complex ligamentous structure responsible to maintain stability on the region.^(2)^

The treatment of these lesions aims to prevent further neurological injury and restore spinal stability.^(2,4,5)^ Especifically dealing with traumatic injuries of the CCJ, multiple fractionated classification systems were proposed to guide treatment, such as those by Anderson et al., for odontoid process fractures,^(6)^ by Effendi et al., for fractures of the posterior elements of the axis arch,^(7)^ and by Traynelis et al., for occipital atlantoaxial dislocation.^(8)^ Some of the classic systems were proposed prior to the advent of modern computed tomography (CT) and magnetic resonance imaging (MRI), which may have precluded a more detailed morphological characterization of osseous and neural tissues compared with these new radiological methods. More importantly, the lack of more clear and comprehensive guidelines contributes to hamper the decision making process between conservative *versus* surgical treatments.

## OBJECTIVE

To evaluate the correlation between the treatment, the characteristics of the lesions and the clinical outcome of patients with traumatic injuries to the craniocervical junction.

## METHODS

A retrospective case series was performed including patients with CCJ spinal trauma treated from 2010 to 2013 at the Hospital of the *Universidade de Campinas*, Campinas (SP) Brazil. We have excluded patients younger than 18 years old, those who had incomplete medical charts and those with pathological fractures.

**Figure 1:**
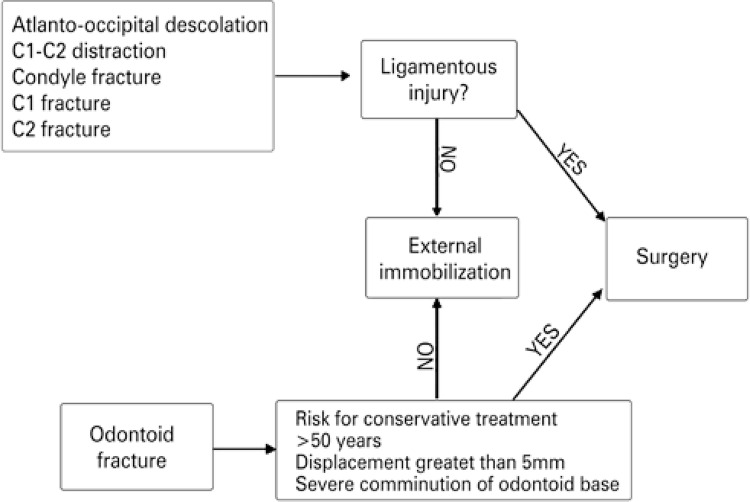
Treatment decision flowchart of patients with lesions in the craniocervical junction (suggested treatment)

Source: Joaquim et al.^(10)^

The clinical and radiological data were evaluated to classify traumas according to patients’ neurological status, injuries morphology and treatment (conservative *versus* surgical treatment). Treatment was performed according to our institution’s algorithm (Figure 1). Ligamentous injuries were referred to early surgical fixation whereas bone fractures without ligamentous injuries were treated according to each injury characteristic (only fracture in the dens base with risk factors for non union were referred for early surgical treatment). Patients were followed after surgery in the outpatient clinic with postoperative CT scan and plain radiographies.

Epidemiological data were presented in a descriptive statistical form and compared with the literature. The specific data were analyzed using the IBM Statistical Package for Social Sciences (SPSS) version 21 for Windows^®^.

The analysis of the groups in relation to the categorical variables used Fisher’s exact test. To compare groups in relation to the numerical variables we used non-parametric Mann-Whitney test. The significance level considered was p≤0.05.

The study was approved by the Research Ethics Committee of the *Faculdade de Ciências Médica da Universidade Estadual de Campinas* number 574.524, CAAE: 24566614.4.0000.5404. A Consent Form was not required.

## RESULTS

We included 43 patients with spinal trauma in the CCJ. Of these, six were excluded: two because of insufficient medical records, one for early death (severe head injury) before any treatment, and three because of being younger than 18 years. Finally, 37 patients were analyzed.

Of the patients, 27 (72.9%) were men. Patients’ age ranged from 20 to 93 years (mean 41.70, standard deviation of 37±16.72 years).

Traffic accidents were the main cause of spinal trauma (59.46%), followed by falls (27.03%).

Initially, 24 patients (64.9%) underwent conservative treatment with rigid cervical collar (Philadelphia), and 12 patients (32.4%) underwent surgical treatment. One patient (2.7%) preseted with severe traumatic brain injury and was referred to later treatment at our institution, with C1-C2 dislocation that was undiagnosed at the service of origin. Patients underwent early surgical treatment when they were at risk for non-consolidation of the fracture, as shown in the algorithm exposed in figure 1.

In the follow-up, seven patients were initially treated conservativelly (29.2%) and underwent late surgery due to treatment failure (non healing in postoperative CT scan after 12 weeks and pain at the fracture site). Among those, six patients presented odontoid fracture in the dens base (all without risk factors for non-union) and one had a non-healed fracture in the posterior elements of the axis. None of these seven patients had delayed neurological *déficits*. All these patients were treated initially with a rigid cervical collar (Philadelphia) and surgery was a posterior C1-2 fixation using screws.

### Initial conservative treatment

**Figure 2:**
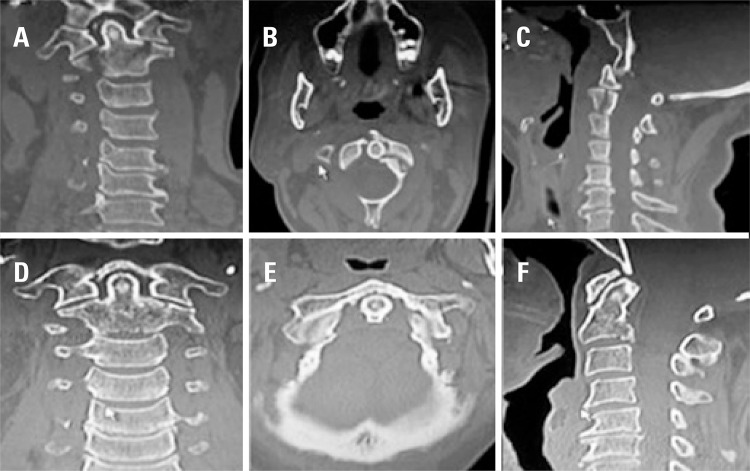
Combined anterior arch of C1 and C2 body fractures - conservative treatment. Line 1: computed tomography images showing the fractures before treatment. (A) coronal, (B) axial, (C) sagittal sections. Line 2: fractures are consolidated after eight weeks of conservative treatment. (D) coronal, (E) axial and (F) sagittal sections

Among 24 patients who initially underwent conservative treatment (Table 1 and Figure 2), five were women (20.8%) and 19 were men (79.1%). Patients’ age ranged from 21 to 93 years old (mean 42.5 years, standard deviation of ± 17.25).

**Table 1:** Classification of the patients who received initial conservative treatment

Injury description	n (%)
Odontoid fracture	
Type II (low risk non-consolidation)	7 (29.2)
Tipe III	1 (4.2)
Hangman fracture	
Type I	2 (8.3)
Type II	3 (12.5)
Occipital condyle fracture	
Type I	2 (8.3)
Type II	2 (8.3)
C1 lateral mass fracture	1 (4.2)
C1 posterior arch fracture	1 (4.2)
C2 body fracture	2 (8.3)
Multiple fractures	
Hangman type I + C1 posterior arch	3 (12.5)
Condyle type I + C2 top facet	
C1 anterior arch + C2 body	

Fractures in a single vertebra were found in 21 patients (87.5%). Three patients had multiple fractures in the spine (12.5%). Among those, one patient (4.2%) had fractures in C1 and C2. Two patientes (8.3%) presented damage in other segments of the spine, in addition to the injuries of the CCJ (T3-T4 and L12).

None of these patients had neurological *déficits* due to the injury of the CCJ. One patient had neurological *déficits* due to spinal cord injury caused by trauma to the thoracic spine (T3-T4). There were no deaths during follow-up nor delayed neurological deterioration. All patients were followed for a minimum period of 8 weeks.

### Failure to initial conservative treatment

Among patients who initially underwent conservative treatment, seven underwent surgery subsequently due to treatment failure. Among these patients, five were men (71.4%). The age distribution was 23 to 64 years, mean 36.5 years, and standard deviation of ±12.95. None of the patients had neurological *déficits*.

**Figure 3:**
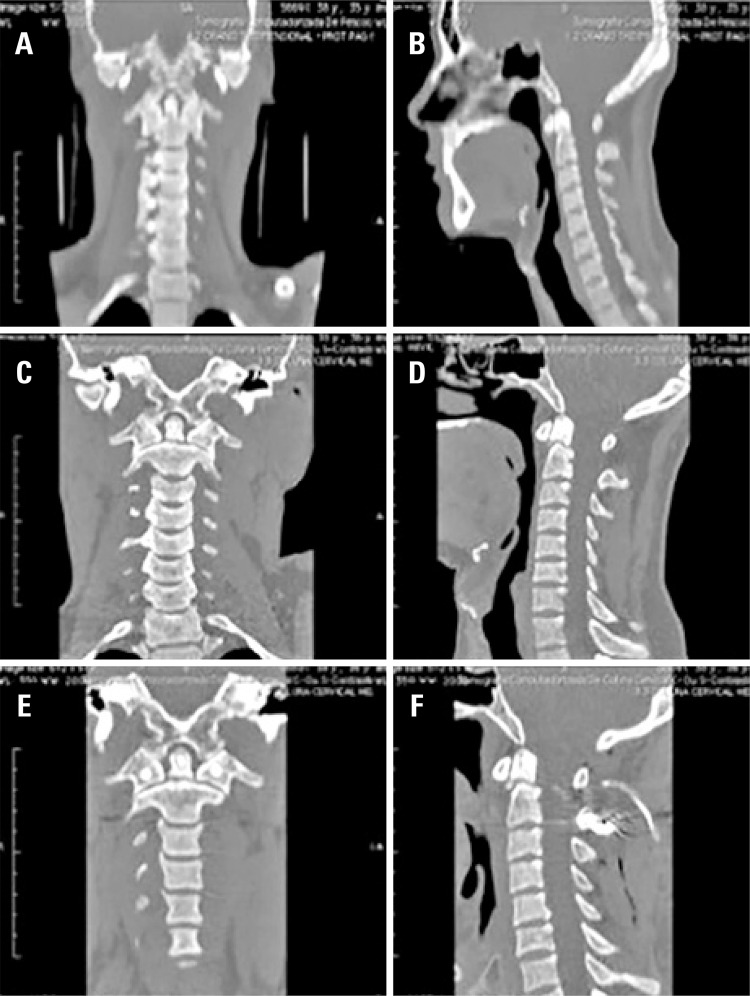
Odontoid fracture type II, conservative treatment failure. (A and B) non-comminuted odontoid fracture without deviation in a 37 years old patient, (C and D) computed tomography scan after eight weeks of conservative treatment showing, (E and F) surgical result after posterior C1-C2 arthrodesis

In our series of seven patients with type II odontoid fractures initially treated conservatively, six underwent surgery subsequently due to non-healing of the fracture. Although conservative treatment was accepted for patients without risk factors for non-consolidation, we noticed that adequate consolidation did not occur in 83% our cases (Figure 3). The high failure rate of conservative treatment for fractures of odontoid type II led us to correlate no healing of the fracture with conservative treatment (p=0.001) (Tables 2 to 4).

### Patients undergoing initial surgical treatment

Two patients had complications (16.6%). One case of fistula treated during surgery, and one case of surgical site infection, which required surgical debridement and antibiotic therapy.

Among the 12 patients initially treated with surgery, only 1 presented pre-operative neurological *déficits* (improving from ASIA C to D during follow-up). There were no instances of new neurological symptoms or death.

All patients were followed for a minimum period of 8 weeks to a maximum of 6 months.

## DISCUSSION

The most frequently found injury in our series was the odontoid fracture, affecting 15 patients (40.54%). Among those, 14 patients had their fracture classified according to Anderson et al., as type II (involving the base of the odontoid process) and one as type III (involving the axis body).^(6)^ Seven cases received indication of early surgical treatment, due to the presence of factors associated with high risk of non-union.^(9,10)^ The remaining patients were treated with immobilization using a Philadelphia collar.

Regarding the treatment of odontoid fractures, Clark et al.,^(11)^ reported the immobilization treatment of types II and III fractures as essential to achieve consolidation. However, consolidation rates in type II fractures with conservative treatment are around 43%, different than type III fractures, in which consolidation is observed in almost 87% of cases.^(11)^

Traynelis et al.,^(12)^ in the largest published study of axis fractures, including 340 cases (199 odontoid fractures) treated with halo vest, obtained 100% healing in type I fractures and 82% in type III. In type II fractures, non-surgical treatment failed in 28% of cases with up to 84% in the case of displacement of the fractured fragment larger than 6mm. They suggested that patients with fracture displacement greater than 6mm should undergo early surgical treatment.

In patients with surgical indication, posterior instrumentation has shown a high rate of arthrodesis.^(13-15)^ In the literature review produced by Julien et al.,^(13)^ 147 patients were retrospectively analyzed with types II and III fractures, obtaining 87% of healing in type II and 100% in type III fractures treated with posterior fixation.

Alternatively, for patients with good bone quality and low risk of postoperative dysphagia, fixation by an anterior approach using an odontoid screw is a reasonable option, with consolidation rates of up to 89% to 100%. This technique has the advantage of preserving mobility between the atlanto-axial joint but it is contraindicated in chronic fractures.^(9,10)^

Finally, in elderly patients, with more than 60 years, several authors^(13,16)^ suggest that the consolidation of fractures with external immobilization is not a good treatment option, since the consolidation rates are generally less than 30%. Controversily, our patients who had failure conservative treatment had a lower mean age (36.5 years) compared with the whole group treated conservatively (42.5 years).

Regarding the use of immobilization by Philadelphia colar or halo vest, Lewis et al.,^(17)^ evaluated 67 patients with odontoid fractures, 32 treated with Philadelphia collar and 37 with halo vest. Consolidation after 3 months was 60% for the group with halo vest *versus* 35% for the group with cervical collar. There were more clinical complications on patients treated with halo vest – 60% *versus* 6% for the group using Philadelphia colar. Despite the differences in bone healing, there was no statistical difference, allowing them to conclude that there was no superiority of one immobilization compared to the other.^(18)^

Despite the high failure rate of conservative treatment, due to its relative low morbidity, it is still a treatment option, once the patient is informed about the likelihood of needing a late surgical procedure and the importance of close clinical and radiological follow-up. After analyzing our results, we are now offering surgery for type 2 odontoid fractures even without risk factors for non-union, explaining the risks and benefits of conservative or surgical treatment. Although we currently advocate surgical fixation, patients should also be consulted in relation to their opinion about the treatment offered.

### Fracture of the posterior elements of the axis

The second most prevalent injury in our series was traumatic spondylolisthesis of the axis, also known as lesions of the posterior elements of the axis or Hangman fracture. There were nine cases (24.32%) in our series.

The treatment of the posterior elements of the axis fracture is relatively well established.^(19,20)^ It is primarily non-surgical, preferably treated with the use of a rigid cervical collar, with surgery reserved for non-healing or deformity or classified fractures as Levine type III. This latter had C2-C3 facet dislocation and ligamentous injuries, which is treated preferentially by surgery in the majority of series already published.^(20,21)^ Anterior or posterior C2-C3 fixation can be used according to surgeon’s preference and injury characteristics.^(9,19,20)^

### Occipital condyle fractures

The occipital condyle fractures were diagnosed in six patients (16.22%). The condylar fracture is probably underdiagnosed because the clinical presentation is variable and it presents no specific signs on physical examination. They are associated with severe head trauma.^(21,22)^

Saternus^(22)^ in a study involving all victims of accidents with injury mechanisms compatible with condyle fracture, found an incidence of 16% of fractures. Literature review conducted by the American Association of Neurological Surgeons^(22,23)^ concluded that not treating condylar fractures is unacceptable. This review identified 23 patients who did not receive treatment: 9 of them had no neurological *déficits* during follow-up. Six other developed late *déficits*, as well as vertigo and nystagmus. In general, with the exception of bilateral fractures associated with atlantooccipital displacement, the condyle fractures can be successfully treated with a rigid cervical collar.^(10,22-24)^

In our series, one patient had bilateral condyle fractures associated with occipital-C1 and C1-C2 dislocation, who underwent an occipital-C2-C3 fixation. There was also a case of condyle fracture associated with lateral mass fracture of C1, which like the other four cases of unilateral fracture of the condyle, underwent conservative treatment with a rigid cervical collar. None of the patients had neurological *déficits* nor worsened during the follow-up.

### Atlas and axis fractures without ligamentous injuries

The atlas fractures occur alone or associated with other fractures. They account for about 1 to 2% of the spinal fractures and 13 to 22% of cervical spine fractures.^(21,23,25)^ These fractures can compromise the anterior, the posterior arch, the lateral mass and the transverse process and may be associated with ligamentous injuries. Thakar et al.,^(26)^ in a prospective series of C1 fractures treated with Philadelfia collar and halo vest, obtained 94% of good results without the need for surgical intervention.

The axis body fractures (no-Hangman) formed a small group. Hadley et al.,^(23)^ reported excellent results with conservative treatment, as surgery should be reserved for cases of burst fracture type or that have other associated injuries.

In our series, there was one case of C1 fracture with facet dislocation treated surgically. The other lesions were successfully treated conservatively with immobilization with a cervical collar. No patient had neurological *déficits* or worsened during follow-up.

Of note, our study is limited by its retrospective nature and because we did not access other confounds factors that may affect bone healing, such as smoking. Additionally, the small number of patients requires caution when interpretating the statisctical analysis.

## CONCLUSION

In our series of traumatic craniocervical junction injuries, odontoid fractures and fractures of the posterior elements of the axis were the most prevalent injuries.

Patients with ligamentous injury were treated successfully with surgery whereas those with isolated bone fractures were preferentially treated with a rigid cervical collar. Our results suggest that early surgery in type II odontoid fractures should be considered due to the high rate of non-consolidation even when factors associated with higher risk for non-union are absent.

**Table 2:** Fractures in patients with conservative treatment failure

Lesion	Total number	Non-healed	(%)
Odontoid fracture type II	7	6	25*
Hangman fracture type II	3	1	4.2^†^
Other injuries	14	0	0

* 85.7% of patients with odontoid fracture type II treated conservatively; ^†^ 33.3% of patients with hangman fracture type II treated conservatively.

**Table 3:** Risk factors according to the treatment performed

Number of risk factors	Therapeutic procedures			Total	p value^*^

	Early surgery	Conservative	Surgery after conservative failure		
None	0	1 (100)	6 (100)	7 (50)	0.001^†^
One	5 (71)	0	0	5 (36)	
Two	2 (29)	0	0	2 (14)	

^*^ Fischer exact test; ^†^ p<0.01.

**Table 4:** Computed tomography *versus* treatment group to odontoid fracture type II

Results	Groups		Total	p value^*^

	Early surgery	Conservative		
Success	7 (100)	1 (14)	8 (57)	0.005
Non-consolidation	0	5 (71)	5 (36)	
Non-consolidation and deviation	0	1 (14)	1 (7)	

Total	7 (100)	7 (100)	14 (100)	

^*^ Fischer exact test; p<0.01.
